# Using Getting To Outcomes to facilitate the use of an evidence-based practice in VA homeless programs: a cluster-randomized trial of an implementation support strategy

**DOI:** 10.1186/s13012-017-0565-0

**Published:** 2017-03-09

**Authors:** Matthew Chinman, Sharon McCarthy, Gordon Hannah, Thomas Hugh Byrne, David A. Smelson

**Affiliations:** 1 VISN 4 Mental Illness Research and Clinical Center and the Center for Health Equity Research and Promotion, VA Pittsburgh Healthcare System, Pittsburgh, PA USA; 2 RAND Corporation, Pittsburgh, PA USA; 3 VA National Center on Homelessness Among Veterans, Philadelphia, PA USA; 4 Boston University School of Social Work, Boston, MA USA; 5 VA Center for Healthcare Organization and Implementation Research, Boston, MA USA; 6 Department of Psychiatry, University of Massachusetts Medical School, Worcester, MA USA

**Keywords:** Implementation support, Co-occurring disorders, Fidelity, Training, Technical assistance

## Abstract

**Background:**

Incorporating evidence-based integrated treatment for dual disorders into typical care settings has been challenging, especially among those serving Veterans who are homeless. This paper presents an evaluation of an effort to incorporate an evidence-based, dual disorder treatment called Maintaining Independence and Sobriety Through Systems Integration, Outreach, and Networking—Veterans Edition (MISSION-Vet) into case management teams serving Veterans who are homeless, using an implementation strategy called Getting To Outcomes (GTO).

**Methods:**

This Hybrid Type III, cluster-randomized controlled trial assessed the impact of GTO over and above MISSION-Vet Implementation as Usual (IU). Both conditions received standard MISSION-Vet training and manuals. The GTO group received an implementation manual, training, technical assistance, and data feedback. The study occurred in teams at three large VA Medical Centers over 2 years. Within each team, existing sub-teams (case managers and Veterans they serve) were the clusters randomly assigned. The trial assessed MISSION-Vet services delivered and collected via administrative data and implementation barriers and facilitators, via semi-structured interview.

**Results:**

No case managers in the IU group initiated MISSION-Vet while 68% in the GTO group did. Seven percent of Veterans with case managers in the GTO group received at least one MISSION-Vet session. Most case managers appreciated the MISSION-Vet materials and felt the GTO planning meetings supported using MISSION-Vet. Case manager interviews also showed that MISSION-Vet could be confusing; there was little involvement from leadership after their initial agreement to participate; the data feedback system had a number of difficulties; and case managers did not have the resources to implement all aspects of MISSION-Vet.

**Conclusions:**

This project shows that GTO-like support can help launch new practices but that multiple implementation facilitators are needed for successful execution of a complex evidence-based program like MISSION-Vet.

**Trial registration:**

ClinicalTrials.gov NCT01430741

## Background

Widespread evidence supports integrating behavioral health and substance abuse treatments for those who are dually diagnosed with both mental illnesses and substance abuse disorders [1]. However, several large-scale initiatives have demonstrated that it is challenging to provide integrated care in typical treatment settings for those who are homeless [2–5]. For example, a multi-site study that integrated dual disorders treatment and Housing First found that while overall scores for Housing First fidelity were high, scores of the item specifically addressing “Integrated, Stage-wise Substance Use Treatment” were rated 3 or lower on a 5-point scale [5]. In the Department of Veterans Affairs (VA), up to 80% of the approximately 48,000 Veterans [6] who are homeless suffer from mental health and/or substance use disorders, threatening their housing stability and leading to higher rates of relapse, treatment dropout, poor community integration, and utilization of costly emergency and inpatient services [7]. This paper presents a cluster-randomized trial of a specific implementation strategy aimed at supporting the use of a complex integrated dual disorders model for Veterans who have experienced homelessness.

Experts in dual diagnosis research have carried out several initiatives attempting to document the degree to which integrated treatments were adopted and implemented. For example, the National Evidence-Based Practice (EBP) Implementation Project was a non-experimental effort to use in-person consultant/trainers for 2 years to help 53 public-sector community mental health agencies adopt two of five EBPs for those with serious mental illnesses (SMI), one of which was an integrated dual disorder model. Although the sites did improve their fidelity, only 18% of the sites who chose the integrated treatment model had high fidelity [2, 3]. Other large-scale initiatives that employed similar implementation strategies found similar results [4, 5]. Building upon these contributions, more research is needed to specifically test implementation strategies in settings that serve those who are not only dually diagnosed but also homeless.

An ideal setting for such research is the VA program called HUD-VASH (Housing and Urban Development—Veteran Affairs Supportive Housing). Veterans at all VA medical centers who are homeless can receive subsidized housing from HUD and VA case management. In 2012, HUD-VASH adopted the Housing First philosophy, which states that individuals do *not* need to demonstrate complete sobriety in order to receive housing and case management services. In addition, HUD-VASH recently accepted a significant number of new Veterans to help support the goal of ending homelessness among Veterans by 2015 [7], straining available resources, especially staff. Both changes increased the number of Veterans in HUD-VASH with a need for integrated treatment for both substance abuse and mental health diagnoses.

Although many evidence-based integrated treatment models are available, one in particular—Maintaining Independence and Sobriety through Systems Integration, Outreach, and Networking—Veterans Edition (MISSION-Vet)—was developed specifically for homeless or formerly homeless Veterans [8, 9]. MISSION-Vet is a manualized, integrated, co-occurring disorder treatment model grounded in the Health Belief Model [10]. In MISSION-Vet, a Veteran, case manager, and “peer specialist” work together for about 2.5 h a week. Peer specialists are individuals who have recovered from their own mental health and substance issues and are trained to provide support to others with the same difficulties [11]. Wide-scale implementation of MISSION-Vet in HUD-VASH has not occurred, despite a strong evidence base, the fact that both share a Housing First treatment philosophy [12], and free, web-based training and manuals. While implementation models such as the Consolidated Framework for Implementation Research (CFIR) [13] show that research efficacy, concrete tools, and compatibility with host sites are important factors that facilitate implementation, these factors alone are usually not sufficient to promote adoption by settings like HUD-VASH [14]. Because systems frequently do not adopt new practices even when they are known to improve outcomes [15], innovative strategies are needed at both individual and organizational levels to encourage adoption.

To facilitate the adoption of MISSION-Vet within HUD-VASH, we employed and then tested the Getting To Outcomes (GTO) approach [16], modified for use within the VA [17]. GTO is both an implementation *model*—specifying steps practitioners should take when carrying out an EBP—and an implementation *strategy*, providing ongoing implementation training, technical assistance, and data feedback to improve practitioners’ capacity to complete those steps [18]. GTO has been found to improve the capacity of individual drug and teen pregnancy prevention practitioners and the performance and fidelity of prevention programs in both quasi-experimental [18] and randomized controlled trials [19–22]. However, most of those studies involved non-evidence-based programs. This study is the first to empirically test a version of GTO in the VA with a rigorous design and also the first instance GTO was used to support an EBP in a clinical setting. We received funding from the VA Quality Enhancement Research Initiative to compare MISSION-Vet implementation with and without GTO support in three large VA HUD-VASH sites.

## Methods

### Overview

This study was a Hybrid Type III, cluster-randomized controlled trial assessing the impact of GTO over and above MISSION-Vet Implementation as Usual (IU). Both conditions received standard MISSION-Vet training and access to the MISSION-Vet treatment manuals, but only the GTO group received implementation support. The study was conducted in HUD-VASH teams at three large VA Medical Centers over a 2-year period (March 2013 to August 2015). Within each HUD-VASH team, existing sub-teams (case managers and Veterans they serve) were the clusters randomly assigned, which is particularly appropriate when desiring to lessen the risk of experimental contamination of the implementation strategy [23]. With support from study staff and technical assistance, each of the GTO groups was able to fund two peer specialists. Necessitated by the scope of the study, this structure represents an adaptation to the MISSION-Vet model, where typically equal numbers of peer specialists and case managers are used to form treatment dyads.

Although Hybrid Type III trials do collect clinical outcome data, they primarily focus on the “utility of an implementation strategy” and measure “adoption of the clinical treatment and fidelity to it, as well as related factors (pg. 220)” [24]. The primary focus of this paper was to evaluate how much the GTO implementation strategy facilitated HUD-VASH’s adoption and implementation of MISSION-Vet, operationalized as MISSION-Vet services delivered. These services were analyzed according to three domains of the implementation heuristic, RE-AIM [25] (Adoption, Reach, Implementation; see below). Blinding data collection was not possible; however, MISSION-Vet service data was from secondary sources, described below. We hypothesized that the GTO group would have greater Adoption, Reach, and Implementation compared to the IU group overall and at each site. To better understand the context for implementation, we interviewed HUD-VASH stakeholders about MISSION-Vet barriers and facilitators based on CFIR. As a Hybrid Type III study, a secondary focus was on the mental health and substance abuse outcomes of the participating Veterans. These were documented by case managers via the VA’s existing HUD-VASH data monitoring system and will be reported in a separate paper.

The VA Central IRB that oversees multi-site trials granted approval for this study in October of 2011. The study received a waiver of consent to use Veteran-level data from the HUD-VASH data management system. Harms of GTO were monitored by study staff during the time span GTO was active. None were reported.

### Participation sites and recruitment

#### Site composition

At the time of the study start, the size of the three HUD-VASH teams were: team 1 (450 Veterans receiving HUD-VASH housing vouchers and 18 case managers), team 2 (850 Veterans receiving HUD-VASH housing vouchers and 27 case managers), and team 3 (810 Veterans receiving HUD-VASH housing vouchers and 24 case managers), for a total of 2110 Veterans receiving HUD-VASH vouchers and 69 case managers. A housing voucher is a subsidy a Veteran receives from HUD that they can use to offset a portion of their rent. Each team is made of two sub-teams of case managers, which were randomized to IU or GTO by the team statistician using a random number generator. The PIs (DS, MC) informed each sub-team of their randomization status. Randomizing within team holds constant the variation due to team-level characteristics, funding streams, regulations, data collection activities, and political climates. The three HUD-VASH teams were selected based on their willingness to participate and their similarity. For example, 85–95% of each team’s vouchers were in active use (Veteran living in an apartment). Data compiled by the VA’s Northeast Program Evaluation Center shows that, on all three teams, all Veterans get at least one contact per month, the HUD-VASH minimum. Approximately 90% of the Veterans on all three HUD-VASH teams have substance use and/or mental health diagnoses according to reports of the VA Homeless Network Coordinators at those sites. Additionally, each of these teams report that approximately 75% of their Veterans receive other services at the VA or in the community beyond standard HUD-VASH case management services.

#### HUD-VASH case manager and Veteran recruitment

The research subjects were the HUD-VASH case managers and peer specialists, but the case managers also recorded data about the Veterans on their caseloads in the VA’s Computerized Patient Record System (CPRS). All case managers were invited to participate and verbally consented before randomization. Ten refused to participate (*n* = 3, 3, and 4 across teams 1–3, respectively); leaving 22 in the IU group and 37 in the GTO group (see Table 3 for team and study condition level sample sizes). A case manager and their total case load of Veterans were assigned to study condition based on the case managers’ team assignment to GTO or IU. All HUD-VASH case managers in both groups were invited to the webinar training on MISSION-Vet. After MISSION-Vet began, the case managers and peer specialists had the opportunity to deliver MISSION-Vet services for up to 1 year to any Veteran who met the following: (1) had a current substance abuse or dependence disorder and a co-occurring mental illness and (2) was willing to receive MISSION-Vet services.

### The evidence-based program: MISSION-Vet

The core service components of MISSION-Vet are critical time intervention (CTI), weekly sessions delivered by a case manager using dual recovery therapy (DRT), and structured and unstructured peer specialist services. The MISSION-Vet Treatment Manual provides guidance to case managers/peer specialists, and the MISSION-Vet Consumer Workbook provides homework assignments, readings, and checklists for Veterans. In several studies, including a randomized matched attention control trial, MISSION-Vet has demonstrated efficacy in increasing treatment engagement, improving mental health and substance abuse outcomes, and reducing ER visits, re-hospitalizations, and recurring homelessness [26–29]. More details about MISSION-Vet are available in this project’s protocol paper published in *Implementation Science* [30].

### GTO implementation model and strategy

Getting To Outcomes (GTO) is a strategy that builds capacity for implementing EBPs by strengthening the knowledge, attitudes, and skills needed to choose, plan, implement, evaluate, and sustain EBPs. GTO helps practitioners address 10 key steps (Table 1) needed to obtain positive results: steps 1–6 for planning EBPs, steps 7–8 for process and outcome evaluation, and steps 9–10 on the use of data to improve and sustain programming.

**Table 1:** Ten GTO steps and information in the manual

GTO step	Manual chapter which….
1	Provides information about conducting a *needs* assessment
2	Has worksheets for creating measurable *goals and objectives*
3	Overviews *evidence-based programming*
4	Ensures programs *fit* with the host agency/other programs
5	Ensures sufficient *capacity* to conduct the program
6	Presents information and tools to *plan* activities
7	Provides information/tools to do *process evaluation*
8	Presents information/tools to do *outcome evaluation*
9	Prompts practitioners to *improve* the program
10	Helps *sustain* an effective program

Italicized text refers to the key word of each GTO step

Implementation of these 10 steps is facilitated by three types of assistance: the GTO manual of text and tools published by the RAND Corporation [31] which was specifically tailored to VA homeless services [32], face-to-face training, and ongoing technical assistance (TA). The goal is to help practitioners and leadership integrate the practices GTO specifies into routine operations and work collaboratively to tailor the EBP to local conditions. Consistent with social cognitive theories of behavioral change [33, 34] and implementation science theories such as CFIR, the GTO supports enhance knowledge about GTO-related activities, which improves attitudes towards these activities, which in turn, improves execution of GTO-specified tasks, which supports the strong implementation of EBP needed for achieving outcomes [35]. For details about how GTO operationalizes CFIR, see Acosta et al. [19] and Smelson et al. [30].

### Use of GTO to facilitate MISSION-Vet in HUD-VASH

Each GTO sub-team created a “GTO Planning Team” of HUD-VASH staff (led by a designated point of contact) and the GTO TA staffperson (SM, in Pittsburgh) who lead the sub-team through the GTO process. The key GTO supports were as follows:

1. *Manual of tools*—each team received the manual *Getting To Outcomes in services for homeless Veterans: 10 steps for achieving accountability* [32], developed from a pilot project at the Pittsburgh VA Homelessness Center [17]. Like all GTO manuals, it provides guidance about how to complete several “tools” or worksheets that prompt practitioners to make, and then record, decisions about various tasks prescribed by the GTO 10 step model.
2. *Training*—study staff (DS, MC, SM) conducted an in-person, 6-h training with each sub-team on how to use GTO to plan, implement, evaluate, and conduct quality improvement on MISSION-Vet. The MISSION-Vet webinar was conducted by DS a few weeks after the GTO training.
3. *GTO technical assistance (TA)*—GTO TA is similar to “facilitation” in the implementation science literature [36]. Like facilitation, GTO TA emphasizes change in work practices through encouragement and action promotion via regular, ongoing meetings [37, 38]. Typically, GTO TA providers guide practitioners to use GTO-based tools to implement an EBP. After feedback from HUD-VASH staff indicated that completing the tools was overly time-consuming, an adaptation was made so that most of the activities that GTO prescribes (and that the tools address) were completed informally during team meetings with the GTO TA staffperson. However, all teams did complete the GTO Goals Tool, which facilitates setting service delivery benchmarks. The TA staffperson met by phone, bi-weekly for 18–23 months with each GTO Planning Team. HUD-VASH team leaders were invited, and those from teams 1 and 2 participated regularly; the team 3 leader participated rarely. As is typical in GTO projects [18, 21, 39], the TA staffperson received implementation supervision via weekly meetings with experts in the EBP (MISSION-Vet, DS) and GTO (MC), respectively. Initial meetings focused on setting goals and performance targets, tailoring MISSION-Vet for HUD-VASH teams, identifying any gaps in skills required by MISSION-Vet, and arranging additional training. Later meetings involved reviewing performance data and troubleshooting implementation. The TA staffperson (SM) visited team 1 twice, team 3 four times, and team 2 only once (for initial training) due to VA travel restrictions. The hours of TA for each team, as recorded in log by the TA staffperson, were 59, 34, and 35 h for teams 1, 2, and 3, respectively. While teams started and ended at slightly different times, they averaged about 1.5 h of technical assistance per month, or about two meetings of 45 min.
4. *MISSION*-*Vet service tracking*—MISSION-Vet service data was collected with a CPRS note template we developed for each team. Data from the notes were extracted to create feedback reports for each sub-team that were discussed in GTO Planning Team meetings approximately once a month.

### Measures and data collection

To document GTO’s impact on MISSION-Vet’s Adoption, Reach, and Implementation, we extracted from the CPRS note templates completed by both groups the following MISSION-Vet services: which DRT sessions, peer specialist sessions, and Consumer Workbook exercises were completed; whether the MISSION-Vet Consumer workbook was provided; whether community activities were done with a Veteran (e.g., taken to appointment, NA/AA meetings, recreational events, meetings with landlords, or other activities); and referrals made to other services. This data was only collected about case managers (and their assigned Veterans) who were consented into the study.

In addition to MISSION-Vet service delivery, we invited all case managers and peer specialists from the GTO group to participate in semi-structured interviews. The response rates for teams 1, 2, and 3 were 83% (*n* = 5), 43% (*n* = 7), 55% (*n* = 10), respectively. Eighteen case managers, six supervisory staff, and one peer specialist were interviewed. The interview protocol was structured around four of the five CFIR domains: Intervention Characteristics, Implementation Process, Inner Setting, and Outer Setting; and the more granular implementation factors under each domain. Similar to Damschroder and Lowery’s use of CFIR to assess context of an implementation [40], we did not collect individual level information appropriate for the Individual Characteristics domain. Questions were drawn from the generic CFIR protocol and then tailored to this project. All interviews were conducted by a doctoral-level researcher who worked on the project (GH) but was not involved in the delivery of the GTO assistance. Interviews involved asking open-ended questions and follow-up probes about how each domain (and implementation factors under each domain) was expressed on the team during MISSION-Vet implementation. All interviews were digitally recorded and transcribed verbatim.

### Data analysis

#### MISSION-Vet services by RE-AIM factors

RE-AIM states that impact of an intervention should be judged by the following factors (http://re-aim.org/): *R*each—the proportion of individuals (in this case, Veterans) who participated in the intervention; *E*ffectiveness—the impact of the program on important outcomes; *A*doption—the absolute number or proportion of clinicians or teams that initiated the intervention; *I*mplementation—the extent to which the interventions’ components were carried out with fidelity; and *M*aintenance—the extent to which a program becomes institutionalized or part of the routine organizational practices and policies. In this report, MISSION-Vet services of both groups were analyzed to assess how much GTO impacted Adoption, Reach, and Implementation—i.e., domains earlier in the cycle of EBP delivery. Table 2 shows how these domains were operationalized.

**Table 2:** RE-AIM-based measures of MISSION-Vet service delivery

RE-AIM domain	RE-AIM indicator	RE-AIM indictor definition
Adoption	Percent of case managers tried MISSION-Vet	The percent of consented case managers who entered at least one MISSION-Vet note
Reach	Percent of case managers who delivered:	The number of unique DRT and Peer sessions received divided by the total number available (13 DRT + 11 peer sessions = 24 available MISSION-Vet sessions)
	any MISSION-Vet sessions	
	10% of MISSION-Vet sessions	
	25% of MISSION-Vet sessions	
	50% of MISSION-Vet sessions	
Implementation	Percent Received workbook	The percentage of Veterans whom had at least one MISSION-Vet CPRS note stating they were given a MISSION-Vet workbook
	DRT sessions done	
	Number	The number of unique DRT sessions given to the Veteran divided by the total possible number of unique DRT sessions (i.e., 13)
	Percent	The number of unique DRT sessions given to the Veteran
	Self-guided exercises done	
	Number	The number of unique self-guided exercises given to the Veteran
	Percent	The number of unique self-guided exercises given to the Veteran divided by the total possible number of unique self-guided exercises (i.e., 7)
	Peer sessions done	
	Number	The number of unique peer sessions given to the Veteran
	Percent	The number of unique peer sessions given to the Veteran divided by the total possible number of unique peer sessions (i.e., 11)
	Number of referrals	The number of unique referral categories (co-occurring, mental health, substance abuse, psychiatric, trauma, housing, vocational, Veteran, medical, and justice,) to which a referral was indicated. Unique referral categories were used rather than absolute number of referrals because review of the data made it clear the staff were frequently reporting the same referral on multiple notes.

For each team, implementation data was collected from the day on which the first MISSION-Vet note was entered until the last day of the month in which the team was formally engaged in the study: team 1 (6/11/13–7/31/14), team 2 (12/31/13–8/31/15), and team 3 (4/19/13–12/31/14). These dates varied due to differences in how long each team participated in the study. For the variables of Adoption and Reach, *Received any MISSION-Vet sessions* were defined as at least one MISSION-Vet session. All the other implementation variables are computed on those Veterans who had at least *two* MISSION-Vet sessions (peer specialist or case manager). This was to exclude Veterans who received the MISSION-Vet orientation session but then chose not to receive MISSION-Vet. Calculations of Reach included all Veterans with a consented case manager. This is a conservative measure of Reach because not all of the Veterans with consented case managers had a dual diagnosis and, thus, were not appropriate candidates for MISSION-Vet. Although data on substance abuse and mental illness were available in CPRS, discussions with case managers suggested that this data was often inaccurate, so all Veterans were included in the denominator to calculate Reach. We used Fisher’s exact test to compare the GTO and Implementation as Usual groups with respect to the Adoption and Reach outcomes. We conducted separate comparisons within each team and across all teams.

#### Ratings of CFIR implementation factors

Based on methods used by Damschroder and Lowery [40], text data from the CFIR-based interviews were used to make ratings for the individual implementation factors under the four domains included. The inclusion and exclusion criteria from the CFIR Codebook Template [41] were used to associate text from the transcripts with CFIR factors. Text could be associated with multiple factors or no factor. One analyst (SM) coded the text initially and a second research associate (GH) reviewed the coding for accuracy. Any discrepancies in the coded text were discussed until consensus was reached. Two analysts then used the text associated with each CFIR factor to rate each factor on the extent to which it impeded or facilitated implementation of MISSION-Vet. Again, discrepancies in the ratings were discussed until consensus was reached. Factors for which there was insufficient text to make a rating were dropped (leaving 20 of 31 CFIR factors). Taking into account both the valence (i.e., either facilitating or hindering) and the strength of each implementation factor, the rating scale ranges from +2 (most facilitating) to −2 (most hindering). Again, discrepancies in ratings were discussed until consensus was reached.

## Results

### MISSION-Vet services by RE-AIM domains

#### Adoption

As shown in Table 3, no case managers in the Implementation as Usual (IU) group at any study site adopted MISSION-Vet while 68% (25 of 37) of case managers in the GTO group adopted MISSION-Vet, a difference that was statistically significant. Likewise, there was a significant difference between the GTO and IU group with respect to the adoption of MISSION-Vet within teams 1 and 2, but not team 3. Teams 1 and 2 had similar rates of case manager Adoption (100, 92%), much more than team 3 (39%). Team 1, which had the highest Adoption among the GTO group (100%), also had the smallest number of consented case managers in the GTO group (*n* = 6). Team 3, which had the lowest Adoption rate among GTO groups (39%), had the largest number of consented case managers (*n* = 18).

**Table 3:** Adoption and reach of MISSION-Vet by study group

	Team 1		Team 2		Team 3		All team	
	GTO	IU	GTO	IU	GTO	IU	GTO	IU
Adoption								
% CM tried MISSION (n)	100* (6)	0 (8)	92* (13)	0 (8)	39 (18)	0 (6)	68* (37)	0 (22)
Reach								
Eligible Veterans^a^, *n*	236	151	271	210	489	206	996	567
Received any MISSION-Vet sessions, % (*n*)	11.4* (27)	0	10.0* (27)	0	3.3 (16)	0	7.0* (70)	0
Received 10% of MISSION-Vet sessions^b^, % (*n*)	7.2* (17)	0	7.4* (20)	0	2.9 (14)	0	5.1* (51)	0
Received 25% of MISSION-Vet sessions^b^, % (*n*)	5.5* (13)	0	4.8* (13)	0	2.2 (11)	0	3.7* (37)	0
Received 50% of MISSION-Vet sessions^b^, % (*n*)	2.5 (6)	0	1.5 (4)	0	1.4 (7)	0	1.7* (17)	0

^a^Eligible Veterans were considered those who had a case manager consented into the study

^b^This is calculated by summing the number of unique DRT and peer sessions received by the total number available (13 DRT + 11 peer sessions = 24 available MISSION sessions)

*Comparison with IU group is significant at the *p* < .05 level based on Fisher’s exact test and Bonferroni-adjusted *p* values

#### Reach

No Veterans with case managers in the IU group (*n* = 567) at any study site received any MISSION-Vet sessions. Seven percent of Veterans with case managers in the GTO group (*n* = 996) received at least one MISSION session. Only 1.7% of Veterans with case managers in the GTO group received at least half of the available 24 structured DRT or peer specialist MISSION-Vet sessions. The differences between the GTO and IU groups with respect to the Reach outcomes were all statistically significant when collapsing across all teams, although not all within-team comparisons were significant. Again, teams 1 and 2 had similar Reach rates, double or more the rates for team 3 across multiple levels of MISSION-Vet received (any, 10%, 25%). As one would expect, the teams with higher Adoption, also had higher Reach (see Table 3). For all teams, the percent of Reach declined as the RE-AIM criterion increased (any, 10%, 25%, 50%).

#### Implementation

Across all teams, 73% of Veterans receiving MISSION-Vet were given the Consumer workbook. Veterans receiving MISSION-Vet received on average 4.2 DRT sessions, 3.3 peer specialist sessions, 1.5 self-guided exercises, and referrals to 1.5 of the categories of referral services. Teams 1 and 3 had similar patterns of case manager implementation (see Table 4). Team 2 provided less case manager services to MISSION-Vet Veterans but more peer specialist services. No team’s Veterans received more than half the sessions of any type called for by MISSION-Vet. Team 1’s Veterans received about a third of the DRT sessions, and a quarter of the self-guided exercises and peer specialist sessions. Team 2’s Veterans received a fifth of the DRT sessions, five percent of the self-guided exercises, and a third of the peer specialist sessions. Team 3’s Veterans received the most: nearly half of the DRT sessions, about a third of the self-guided exercises, and about a quarter of the peer specialist sessions.

**Table 4:** Implementation of MISSION-Vet

Dose level of those who received at least two sessions with either case manager or peer specialist	Team 1 (*n* = 24)		Team 2 (*n* = 24)		Team 3 (*n* = 18)		All (*N* = 66)
	*M*	Range	*M*	Range	*M*	Range	*M*
Percent received workbook	79	–	63	–	78	–	73
DRT sessions done							
Number	4.5	0–11	2.6	0–10	6.0	0–13	4.2
Percent^a^	34	0–85	20	0–77	46	0–100	32
Self-guided exercises done							
Number	1.7	0–6	0.4	0–3	2.7	0–7	1.5
Percent^b^	24	0–86	5	0–43	38	0–100	21
Peer specialist sessions done							
Number	2.8	0–11	4.0	0–10	2.9	0–8	3.3
Percent^c^	25	0–100	36	0–91	27	0–73	30
Number of referrals	1.6	0–6	1.1	0–4	2.1	0–6	1.5

^a^Percentage of 13 DRT sessions

^b^Percentage of 7 self-guided exercises

^c^Percentage of 11 peer sessions

### Ratings of CFIR implementation factors

In this study, the domain of Intervention Characteristics addresses characteristics of the MISSION-Vet model (see Table 5). All teams perceived that the decision to use the MISSION-Vet model was reached externally to HUD-VASH (Intervention source), which is typically an implementation barrier. Teams 1 and 3 found MISSION-Vet to be complicated to implement while team 2 was neutral on Complexity. Across all teams, MISSION-Vet was perceived as highly adaptable, such that case managers could implement different aspects of MISSION-Vet in different ways for different Veterans, and this was seen as a major facilitator because of the flexibility it afforded. Further, all teams rated Relative Advantage as a facilitator, as case managers reported that MISSION-Vet offered concrete, useful ideas for how to treat those with dual diagnoses that were good additions to their current strategies.

**Table 5:** CFIR ratings of study teams

CFIR constructs	Team 1	Team 2	Team 3
Intervention characteristics			
Intervention source	−1	−1	−1
Evidence strength	0	0	0
Relative advantage	+1	+1	+1
Adaptability	+2	+2	+2
Complexity	−1	0	−1
Design quality	0	−1	0
Inner setting			
Networks and communications	−2	+1	−1
Compatibility	−1	−2	−1
Relative priority	−2	−2	−2
Org. incentives and rewards	−1	0	−1
Goals and feedback	0	0	−1
Leadership engagement	−1	+1	−1
Available resources	−1	−1	+1
Access to knowledge and info	−1	0	+1
Outer setting			
Patient needs and resources	+1	+1	+1
Cosmopolitan	0	0	+1
Process			
Planning	+1	+1	+1
Engaging key stakeholders	+1	0	+1
Engaging Veterans	−1	−2	−1

The CFIR Inner Setting domain refers to the setting where the intervention took place, in this study, the HUD-VASH teams. At this level, the low relative priority given to implementing MISSION-Vet by the teams’ leadership, in comparison to the high pressure to house Veterans quickly, was seen as a major barrier to implementation across all three study teams. Further, many case managers found MISSION-Vet was incompatible with their work on the HUD-VASH team, as urgent pressure to house Veterans, and large caseload sizes left case managers with little time for the intensity of providing MISSION-Vet services. Case managers reported no positive influence from Incentives or Rewards by leadership for using MISSION-Vet (negative at two teams, neutral at another) or by any feedback provided about use of MISSION-Vet (neutral at two teams, negative at another). Team communications were also challenging at two of the three sites, especially so for the one site that hired peers specialists via contract (not as VA employees), which added an additional level of communication complexity.

There was only sufficient data available to provide ratings for two constructs in the Outer Setting domain. Understanding of patient needs and resources across all three teams were a positive factor in implementation. However, staff at team 2 made repeated statements indicating their belief that the MISSION-Vet intervention was inappropriate for the population they served because their Veterans were struggling to meet basic needs (housing, food, and clothing) and were not ready to address issues related to their dual diagnosis. This perception was seen as negatively affecting the implementation of MISSION-Vet on this team.

In the Process domain, planning for implementation—operationalized by the GTO implementation strategy—was seen as a strength—as GTO helped organize the teams, kept them on track, and provided a forum to troubleshoot implementation. All teams reported significant challenges engaging Veterans into MISSION-Vet treatment, which is reflected in the negative rating on the Engaging Veterans factor.

## Discussion

We presented data assessing the impact of an implementation strategy (GTO) on the use of a clinical treatment (MISSION-Vet) in three domains specified by the implementation heuristic, RE-AIM (Adoption, Reach, Implementation). To provide additional context, we also assessed various factors that could hinder or facilitate implementation according to CFIR. As hypothesized, the GTO group had greater rates of Adoption, Reach, and Implementation than the IU group. Regarding Adoption, most case managers assigned to GTO attempted MISSION-Vet, while no case managers in the IU group did. Given that case managers in the IU group had zero Adoption, they also had zero Reach and Implementation. These findings are consistent with a great deal of research that shows that passive approaches to implementation do not usually result in uptake of new practices [42]. Although case managers in the GTO condition attempted to deliver MISSION-Vet more than IU case managers, they only implemented MISSION-Vet with a small number of Veterans (Reach) and delivered a small amount of MISSION-Vet services compared to what the EBP typically requires (Implementation). The findings regarding the low level of implementation are different from past GTO studies [22] but similar to past studies attempting to facilitate the delivery of integrated dual diagnosis treatment, as described above [2–5]. Compared to MISSION-Vet, however, the EBP in GTO studies have been much less complicated to implement—e.g., an eight-session teen pregnancy prevention program [22]. As we show below, the CFIR ratings highlight the numerous challenges on the teams that made the use of MISSION-Vet more difficult and some factors which aided implementation.

### Intervention characteristics

Although most case managers appreciated the guidance and materials of MISSION-Vet, some case managers found the treatment complicated and confusing. In particular, some stated that it was challenging to know when and how to integrate the various components of MISSION-Vet into the treatment for each Veteran. MISSION-Vet was adapted quite often, which facilitated case managers trying it out, but appeared to have made it “acceptable” to not implement it in full.

### Inner setting

There were several factors associated with the HUD-VASH setting that made MISSION-Vet implementation more challenging. First, although approval for introducing MISSION-Vet was secured from both medical center and HUD-VASH levels, there was little leadership involvement from either level once the project began. We primarily worked with frontline staff to implement MISSION-Vet. Without higher level leadership buy-in, there was little accountability when implementation lagged or reward for those who did implement. Through GTO, service delivery goals were established, but leadership did not track performance compared to those goals. The teams recognized that many Veterans needed more comprehensive dual diagnosis treatment; however, there were mixed feelings about whether it was reasonable for case managers to deliver this treatment given constant pressure to meet goals of housing Veterans within specified time frames. MISSION-Vet is a comprehensive treatment that requires 2.5 h a week of clinical time, which was incompatible with the increasingly large case load size of the HUD-VASH case managers.

Another significant challenge was that the data feedback system established through the GTO strategy experienced a number of difficulties which undercut its effectiveness and impact. First, case managers and peer specialists did not always use the note template, and it is likely that more services were delivered than recorded for many of those staff. Second, the VA data infrastructure made capturing the data extremely difficult and required searching, accessing, and merging records across multiple systems. Although the data was checked for errors, it is likely that some data errors remained. Third, because the VA system did not have an easy mechanism to capture the MISSION-Vet service data, there were often discrepancies between what case managers reported they were delivering and what the data reports stated. All together, these circumstances made the resulting feedback reports less accurate and thus the case managers were mixed on the utility of the feedback reports.

### Outer setting

Serving patients was a priority for HUD-VASH case managers, and that facilitated trying MISSION-Vet according to the CFIR ratings. Although not officially rated, two other factors in the CFIR Outer Setting domain, Peer Pressure (competition between teams), and the presence of external policies and incentives may have been working against implementation. That is because there was not a large-scale effort within VA to use MISSION-Vet within HUD-VASH—i.e., there was little national momentum for its implementation. Thus, there were no external policies calling for MISSION-Vet across the VA nor were there any external incentives to deploy it. In the context of President Obama’s pledge to end Veteran homelessness by 2015, the incentives were tightly aligned to securing housing.

### Implementation process

Ongoing planning meetings of the GTO teams at each site helped keep the HUD-VASH case managers focused on trying out MISSION-Vet. The GTO technical assistance staffperson, who ran those meetings, was the external change agent. However, case managers reported great difficulty in engaging Veterans to try MISSION-Vet. As stated, MISSION-Vet is intensive, and many Veterans in HUD-VASH who have substance abuse disorders may not have been ready to engage in such an intensive treatment.

### Limitations

Certain limitations should be noted. First, peer specialists were not available to teams in the IU condition in two of the three sites. The activation provided by GTO facilitated peer specialists being added to the intervention (GTO) teams, but the roll out of peer specialists varied: one team used contract peer specialists which involved greater complexity and coordination, one team had significant turn over in their peer specialists during the study, and one team experienced challenges in peer specialists recording notes into the templates. In addition, the shortage of peer specialists in this study meant that the case managers in all three GTO teams had to “share” peer specialists, which is not typical for MISSION-Vet implementation. Second, we were able to interview only one of the eight peer specialists who had been involved over time at the three teams. Third, the data from the electronic medical record were limited and relied upon case managers, not trained data collectors. It was not possible to completely confirm accuracy of the data, although we were able to make some data corrections by getting feedback from the case managers (via the data feedback process). Fourth, we know that the services documented underrepresented the actual amount of services delivered. Case managers and peer specialists in all GTO sites reported delivering some services not recorded on the CPRS note template. Lastly, using all Veterans on the HUD-VASH sub-teams as the denominator for calculation of Reach likely made Reach rates appear lower and could be considered a “lower bound” for Reach. That is because not all HUD-VASH Veterans have a dual diagnosis and would be appropriate for MISSION-Vet services.

## Conclusions

This study suggests that while GTO implementation support was critical to the launch of MISSION-Vet, this alone does not equate to service delivery with high fidelity for a complex intervention like MISSION-Vet. Despite the support for the launching of MISSION-Vet services, a constellation of implementation factors on the ground (highlighted by the CFIR model) exerted tremendous influence on the amount of MISSION-Vet delivered and consequently reduced fidelity. This project was launched right as the VA was tasked to reduce the number of Veterans who were homeless to zero and HUD-VASH had been asked to adopt a Housing First philosophy (accepting Veterans into services much earlier in their recovery). As a result, case managers were under a great deal of pressure to house more Veterans who had more significant impairments than ever before, which left little room to take on new initiatives, regardless of their utility. It is possible that through the GTO approach, more could have been done to engage leadership levels beyond the team at each site; however, in the absence of any national momentum or external policies, it was unclear what incentives to leverage.

It could be concluded that MISSION-Vet was not an optimal fit for the HUD-VASH program at the time this study was conducted. Nonetheless, while choice in service engagement is a core philosophy for homeless clients in Housing First programs, it is also important to address co-occurring metal health and substance abuse to prevent housing instability and loss, an area with which the field is still struggling to optimally deliver support [43]. Thus, given its evidence base and philosophical overlap with HUD-VASH, it is possible that under different circumstances, MISSION-Vet could be successful. Since the end of the trial, the national HUD-VASH program has funded additional training for Critical Time Intervention (a key component of MISSION-Vet), multidisciplinary teams, and shared caseloads as a way to improve access to a variety of services including integrated dual disorders treatment. As the study ended, a GTO team at one of the sites was in the process of creating a special intensive case management sub-team in which case managers with very low caseloads would use MISSION-Vet to treat high-acuity patients. This idea appears to be more favorable as it has high leadership support, has high priority, and would be more compatible with the new case load configuration—CFIR factors that were implementation barriers during the study. Overall, this project shows that GTO-like support can help launch new practices but that multiple implementation facilitators are needed for successful implementation of an evidence-based program like MISSION-Vet.

## Abbreviations

CPRS: Computerized Patient Record System
EBP: Evidence-based practice
GTO: Getting To Outcomes
HUD-VASH: Housing and Urban Development—Veteran Affairs Supportive Housing
MISSION-Vet: Maintaining Independence and Sobriety through Systems Integration, Outreach, and Networking—Veterans Edition
